# Targeting BCL‑2 through Deep Learning-Based Drug Repurposing: A Multimodal Approach Combining Diffusion-Based Generative Modeling, Neural Relational Inference, and In Vitro Validation

**DOI:** 10.1021/acs.jcim.6c01299

**Published:** 2026-08-14

**Authors:** Ehsan Sayyah, Hüseyin Tunç, Asuman Çelebi, Timuçin Avşar, Serdar Durdağı

**Affiliations:** † Lab for Innovative Drugs (Lab4IND), Computational Drug Design Center (HİTMER), 52946 Bahçeşehir University, İstanbul 34349, Türkiye; ‡ Department of Biostatistics and Medical Informatics, School of Medicine, Bahçeşehir University, Istanbul 34349, Türkiye; § Department of Medical Biology, School of Medicine, Bahçeşehir University, Istanbul 34349, Türkiye; ∥ Molecular Therapy Lab, Department of Pharmaceutical Chemistry, School of Pharmacy, Bahçeşehir University, Istanbul 34353, Türkiye; ⊥ Quantitative System Biology Lab, Faculty of Medicine, Biruni University, İstanbul 34010, Türkiye

## Abstract

Accurate identification of repurposable BCL-2 ligands requires not only plausible bound complex structures but also a dynamic description of how ligand binding reshapes residue-level communication. Here, we present a multimodal BCL-2 repurposing workflow built with diffusion-based generative modeling for ligand-specific complex generation and an extended neural relational inference (NRI) framework for trajectory-level interaction analysis. NeuralPlexer was applied to a library of 3094 FDA-approved drugs to generate BCL-2-ligand complex conformations at scale, yielding 1294 structurally acceptable complexes for downstream prioritization. To complement static scoring, filtered candidates were evaluated by molecular docking, anticancer QSAR classification, all-atom molecular dynamics (MD) simulations, and MM/GBSA binding free-energy calculations. We then extended NRI to protein–ligand trajectories to quantify residue-ligand and residue–residue dynamic couplings, enabling comparison of candidate-specific interaction signatures against the reference BCL-2 inhibitor Venetoclax. Among the prioritized compounds, Relugolix emerged as one of the most compelling hits, combining favorable binding energetics with an NRI-derived interaction pattern closely resembling that of Venetoclax. *In vitro* experiments supported BCL-2 inhibition by Relugolix in a TR-FRET assay and reduced viability of LN-18 glioma cells (IC_50_ = 23.55 μM). Together, these results establish a strategy that couples generative complex prediction with graph-based dynamic inference for structure-guided drug repurposing and identify Relugolix as a tractable scaffold for future BCL-2 inhibitor design.

## Graphical abstract

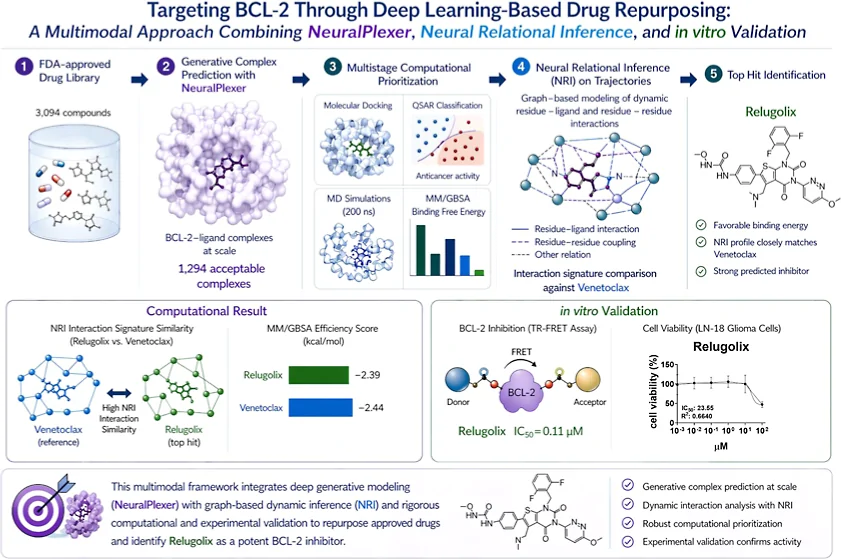

## 1 Introduction

The B-cell lymphoma 2 (BCL-2) family plays a central role in the regulation of apoptosis, and dysregulation of this pathway is a hallmark of many cancers.^1–6^ Overexpression of antiapoptotic proteins such as BCL-2 and BCL-xL enables malignant cells to evade programmed cell death and contributes to therapeutic resistance.^1,3–6^ As a result, BCL-2 has become a clinically validated target for anticancer drug discovery, most notably through the development of BH3-mimetic inhibitors such as Venetoclax.^1,2,7–9^ Although Venetoclax has demonstrated major therapeutic benefit, particularly in hematologic malignancies, the continued need for compounds with improved selectivity, broader applicability, and favorable safety profiles has sustained interest in the discovery of novel BCL-2-targeting ligands.^1,3,5–9^

Drug repurposing offers an attractive strategy for accelerating this process because approved drugs already possess substantial pharmacological and safety information.^10,11^ In the context of BCL-2, repurposing may enable rapid identification of chemically diverse scaffolds capable of modulating apoptosis-related signaling. However, successful repurposing remains challenging because accurate prioritization depends on more than ligand similarity or static binding scores. In particular, the binding pose of the ligand within the BCL-2 binding groove, the induced structural adaptation of the target protein, and the resulting residue-level interaction network can all influence whether a candidate behaves as a productive binder. Thus, computational workflows that can capture both plausible bound structures and their dynamic consequences are especially valuable for repurposing studies targeting BCL-2.

Traditional target-based screening methods, including molecular docking and molecular dynamics (MD) simulations, remain widely used for protein–ligand modeling, but they carry important limitations. Docking methods rely on simplified sampling schemes and approximate scoring functions, which can inadequately represent structural cooperativity between ligand binding and receptor rearrangement. Although MD simulations provide full time-dependent trajectory information, their practical application in large-scale screening is constrained by substantial computational cost and the need for extensive sampling to approach convergence. Consequently, standard MD-based workflows are most informative when applied to a focused set of preselected candidates rather than to large compound libraries. By contrast, recent deep generative models provide an opportunity to predict protein–ligand complexes in a more state-aware manner. Such as NeuralPlexer is a multiscale generative framework that predicts protein–ligand complex structures directly from protein sequence and ligand graph representations.^12–14^ By combining residue-level contact prediction with progressive all-atom refinement and diffusion-based ligand coordinate generation, NeuralPlexer can model bound complexes while incorporating key biophysical constraints such as bond geometry and steric compatibility.^12–14^ These features make it particularly attractive for large-scale generation of ligand-specific complex conformations prior to downstream energetic analysis. NeuralPlexer captures the structural cooperativity between ligand binding and protein conformation, overcoming a major limitation of conventional docking methods that often treat the receptor as rigid. Using a diffusion-based generative process, it iteratively samples diverse ligand poses while enforcing key biophysical constraints, including bond lengths, bond angles, and steric compatibility. By incorporating these priors directly into model generation, NeuralPlexer produces binding-site conformations that are both geometrically plausible and energetically favorable. Its performance has been extensively benchmarked in tasks such as flexible binding-site structure recovery and blind protein–ligand binding.^12–14^ In these evaluations, NeuralPlexer has consistently outperformed state-of-the-art approaches, including AlphaFold2, in both global structure accuracy and the prediction of ligand-induced conformational changes.^12–14^

Even when plausible bound complexes have been generated and MD simulations have been performed, prioritization reduced to a single end point conformation, such as the lowest-energy docked pose or a trajectory end-state, may fail to capture the time-dependent fluctuations and ligand-induced conformational changes that govern binding stability. Approaches that extract and integrate interaction patterns from MD trajectories across many frames, therefore, provide a level of mechanistic discrimination that single-structure or single-point energetic scoring alone may not achieve. Neural relational inference (NRI) is a graph-based variational framework that learns latent interaction maps from dynamical systems and has recently been applied to protein motions.^15,16^ In its original form, NRI was used primarily to study residue–residue communication in proteins. Here, first time in literature, NRI was incorporated to go beyond per-residue contact frequency analysis. By treating each residue as a node in a latent interaction graph and learning edge types from trajectory data, NRI can detect noncovalent interaction channels, including allosteric communication pathways, that remain invisible to conventional distance-based contact maps. This is particularly relevant for BCL-2, where induced-fit rearrangements of the hydrophobic groove upon ligand binding are mechanistically important and not fully captured by pairwise distance cutoffs alone. Such an approach could help distinguish compounds that merely occupy the binding pocket from those that reproduce a more functionally relevant dynamic interaction architecture.

Thus, in this study, we developed a multimodal repurposing workflow centered on diffusion-based generative model complex generation and an extended NRI analysis of protein–ligand MD trajectories to identify FDA-approved compounds with potential BCL-2 inhibitory activity. A library of 3094 approved drugs was screened by NeuralPlexer to generate ligand-specific BCL-2 complex conformations. This process yielded around 1300 successful binding poses, representing individual 3D conformations of BCL-2 for each bound ligand. Subsequently, we calculated the interaction energies (i.e., score-in-place) for these protein–ligand poses, which were then filtered and prioritized using docking refinement, anticancer Quantitative Structure–Activity Relationships (QSAR) classification, MD simulations, and Molecular Mechanics/Generalized Born Surface Area (MM/GBSA) binding free-energy calculations. In parallel, a Chemprop-based pIC_50_ prediction model^17^ and GNINA docking^18^ were used as an orthogonal ligand-centric prioritization arm. We then applied the extended NRI framework to characterize residue-ligand and residue–residue dynamic couplings and to compare candidate interaction signatures against Venetoclax. This integrated strategy prioritized the most compelling hit compounds, and subsequent Time-Resolved Fluorescence Resonance Energy Transfer (TR-FRET) and cell-viability assays provided initial validation of selected hit compounds as potential BCL-2 inhibitors. To the best of our knowledge, this work represents the first application of diffusion-based generative modeling to large-scale screening of an FDA-approved drug library against BCL-2. Collectively, our findings illustrate how generative complex prediction and graph-based dynamic inference can be combined to support structure-guided drug repurposing for BCL-2-driven malignancies.

## 2 Results

### 2.1 NeuralPlexer-Guided Structure-Based Prioritization of FDA-Approved BCL-2 Candidates

In this study, we performed an *in silico* analysis to identify FDA-approved compounds that show promise in targeting the BCL-2 protein, an important apoptosis regulator and a desirable target for cancer treatment. Utilizing the cutting-edge protein structure prediction model NeuralPlexer, we produced a library of 3094 FDA-approved compounds as well as precise and sensitive conformations of the BCL-2 protein. NeuralPlexer derived 8 conformations for every protein–ligand interaction using the whole sequence of the BCL-2 protein and then ranked them according to confidence scores. Therefore, we were able to focus on the most promising binding positions for additional examinations. The protein–ligand complexes obtained for each BCL-2/ligand complex were re-scored by Glide SP (Standard Precision) with the “refine only” option, which optimizes the ligand conformation without requiring any sampling in docking. This method enabled efficient evaluation of the binding of FDA-approved drugs within the BCL-2 binding site. The initial screening library contained 3094 FDA-approved compounds obtained from DrugBank. For each compound, NeuralPlexer generated up to eight BCL-2–ligand complex conformations, and the highest-confidence poses were evaluated for structural acceptability based on pose geometry and model confidence. This first filtering step retained 1294 structurally acceptable successful BCL-2 ligand complexes for downstream rescoring. These complexes were then subjected to Glide SP scoring in “refine only” mode, which locally optimized the NeuralPlexer-generated ligand pose without performing full redocking. To ensure that Glide refinement did not substantially alter the original NeuralPlexer binding mode, only complexes with a ligand RMSD <2 Å between the NeuralPlexer-generated and Glide-refined poses were retained, yielding 463 compounds. These 463 compounds were ranked according to their Glide SP docking scores, and the 20 compounds with the most favorable scores were selected for further filtering. Finally, the top-20 compounds were evaluated using the MetaCore anticancer binary QSAR model, and compounds with anticancer activity scores >0.5 were advanced, resulting in 10 final candidates for all-atom MD simulations, MM/GBSA binding free-energy calculations, and NRI-based trajectory analysis. Thus, the final compound set was obtained through a sequential, stage-gated prioritization strategy rather than by a single docking or scoring criterion.

Importantly, compound prioritization in this workflow was implemented as a sequential, stage-gated decision process rather than as a single-score ranking procedure. NeuralPlexer was first used to generate ligand-specific BCL-2 complex conformations, and only structurally acceptable poses were retained for downstream assessment. Glide SP “refine only” scoring was then applied to evaluate whether the NeuralPlexer-generated binding geometries could be locally optimized while preserving the predicted pose, with RMSD filtering used to exclude complexes that underwent substantial pose rearrangement during refinement. The retained compounds were subsequently filtered by predicted anticancer activity, followed by MD simulations and MM/GBSA calculations to evaluate dynamic stability and relative binding energetics. Thus, molecular docking and MM/GBSA were used as prioritization and enrichment tools, whereas the final candidate selection was based on convergence across structural plausibility, pose stability, predicted anticancer relevance, MD/MM/GBSA behavior, NRI-derived dynamic interaction signatures, and experimental validation.

Figure 1 shows a violin plot of the docking score distribution for all 1294 compounds, together with the fractions of molecules exhibiting RMSD values above and below 2 Å. The 1294 docked compounds were then ranked according to their Glide scores, and the top-20 scored candidates were selected for further analysis. These compounds were subsequently evaluated using a binary QSAR model in MetaCore (https://portal.genego.com/) to predict anticancer activity.^19^ Among the top-20 compounds, 10 of them with predicted anticancer activity scores greater than 0.5 and favorable Glide scores were retained for subsequent studies. These selected candidates were subjected to 3 independent replicates of 200 ns all-atom MD simulations in Desmond, and their average binding free energies were estimated using the MM/GBSA method. For comparison, the same MD and MM/GBSA analyses were also carried out for Venetoclax, the FDA-approved BCL-2 inhibitor used as a reference compound.

**Figure 1:**
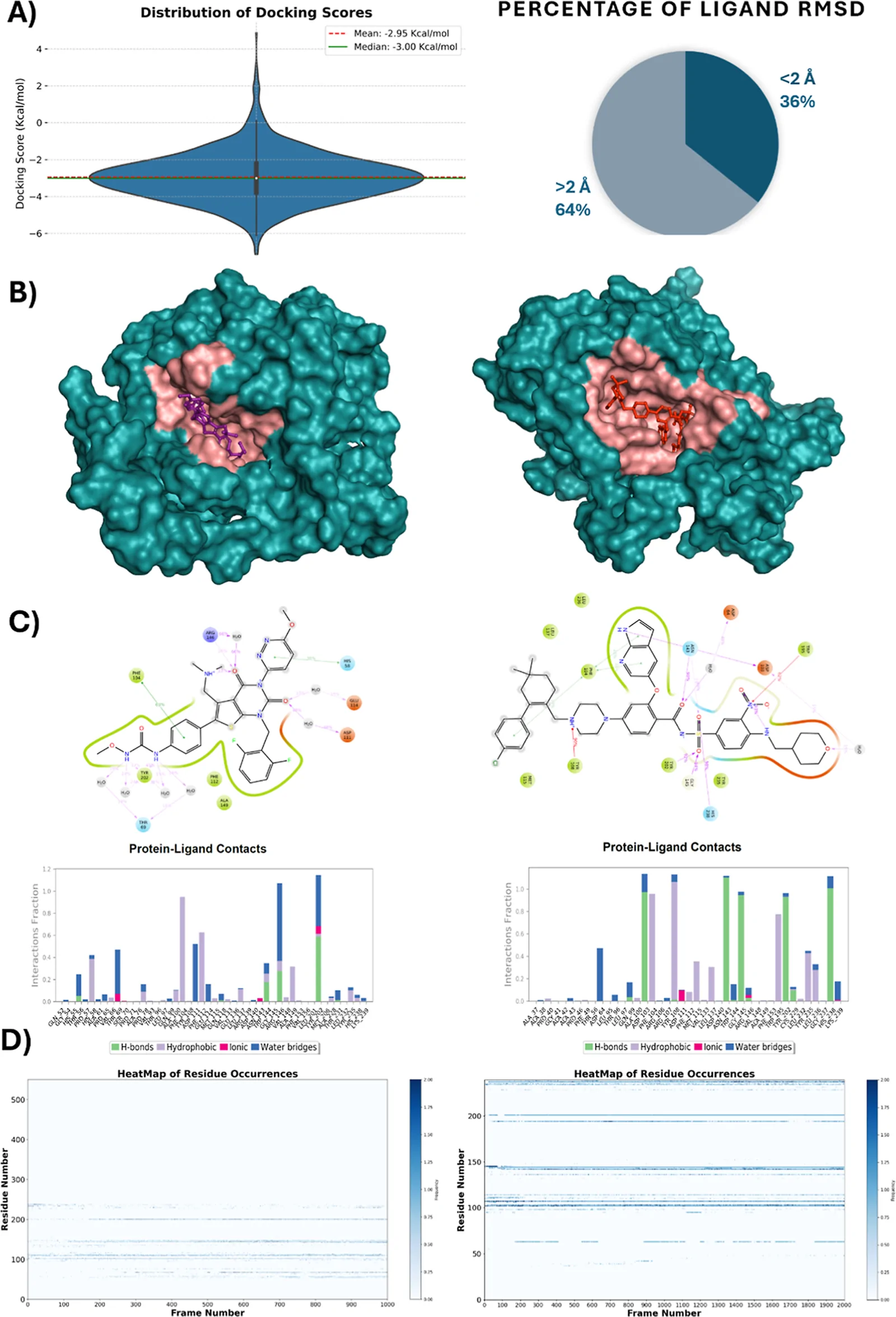
(A) The violin plot of the distribution of all 1294 molecules with the score-in-place docking score and the percentage of molecules with RMSD over and under 2 Å; (B) 3D structure of the BCL-2 protein in complex with Venetoclax on the left and Relugolix on the right; (C) the interaction types of Venetoclax and Relugolix in the left and right, respectively, during the 200 ns MD simulations; (D) the heat map of the BCL-2 residues interacting with Venetoclax on the left and Relugolix on the right.

Venetoclax showed the most favorable average MM/GBSA binding free energy (−132.14 kcal/mol), exceeding those of the top candidate compounds identified in this study. However, because Venetoclax is relatively large (molecular weight 868 g/mol), MM/GBSA values were additionally normalized on a per-non-hydrogen-atom basis to reduce size-related bias and enable comparison in terms of ligand efficiency. Ligand efficiency, which reflects binding energy relative to molecular size, is a useful metric in drug discovery because compounds with favorable efficiency often display more balanced drug-like properties. Based on this analysis, only Lathyrol may not show better ligand efficiency than Venetoclax. The corresponding ligand efficiency value of Lathyrol is −1.87 kcal/mol, compared with −2.17 kcal/mol for Venetoclax. These results suggest that, despite weaker average MM/GBSA binding energies than Venetoclax, these compounds may engage the BCL-2 binding site efficiently relative to their molecular size. All scores for the 10 FDA-approved compounds and Venetoclax are summarized in Table 1, and their 2D structures are shown in Figure 2.

**Table 1:** FDA-Approved Compounds Obtained by NeuralPlexer with Venetoclax as the Reference Molecule, along with Their 3-Replicate Average MM/GBSA Scores, Docking Scores, Anticancer Activity Scores, and Ligand Efficiency Statistics

compound	average MM/GBSA (kcal/mol)	docking score (kcal/mol)	anticancer activity score	ligand efficiency (kcal/mol)
Venetoclax	–132.14	-^a^	0.72	–2.17
Chlorzoxazone	–34.84	–4.83	0.86	–3.17
Etofylline	–46.09	–5.15	0.83	–2.88
Ospemifene	–76.10	–5.27	0.79	–2.82
Vericiguat	–78.19	–5.38	0.77	–2.52
Fadrozole	–41.88	–5.52	0.5	–2.46
Ensulizole	–46.09	–5.41	0.79	–2.43
Doxifluridine	–40.90	–4.96	0.9	–2.41
Relugolix	–103.86	–4.98	0.57	–2.36
Perospirone	–68.32	–6.96	0.54	–2.28
Lathyrol	–44.99	–5.12	0.58	–1.87

a No docking score for Venetoclax.

**Figure 2:**
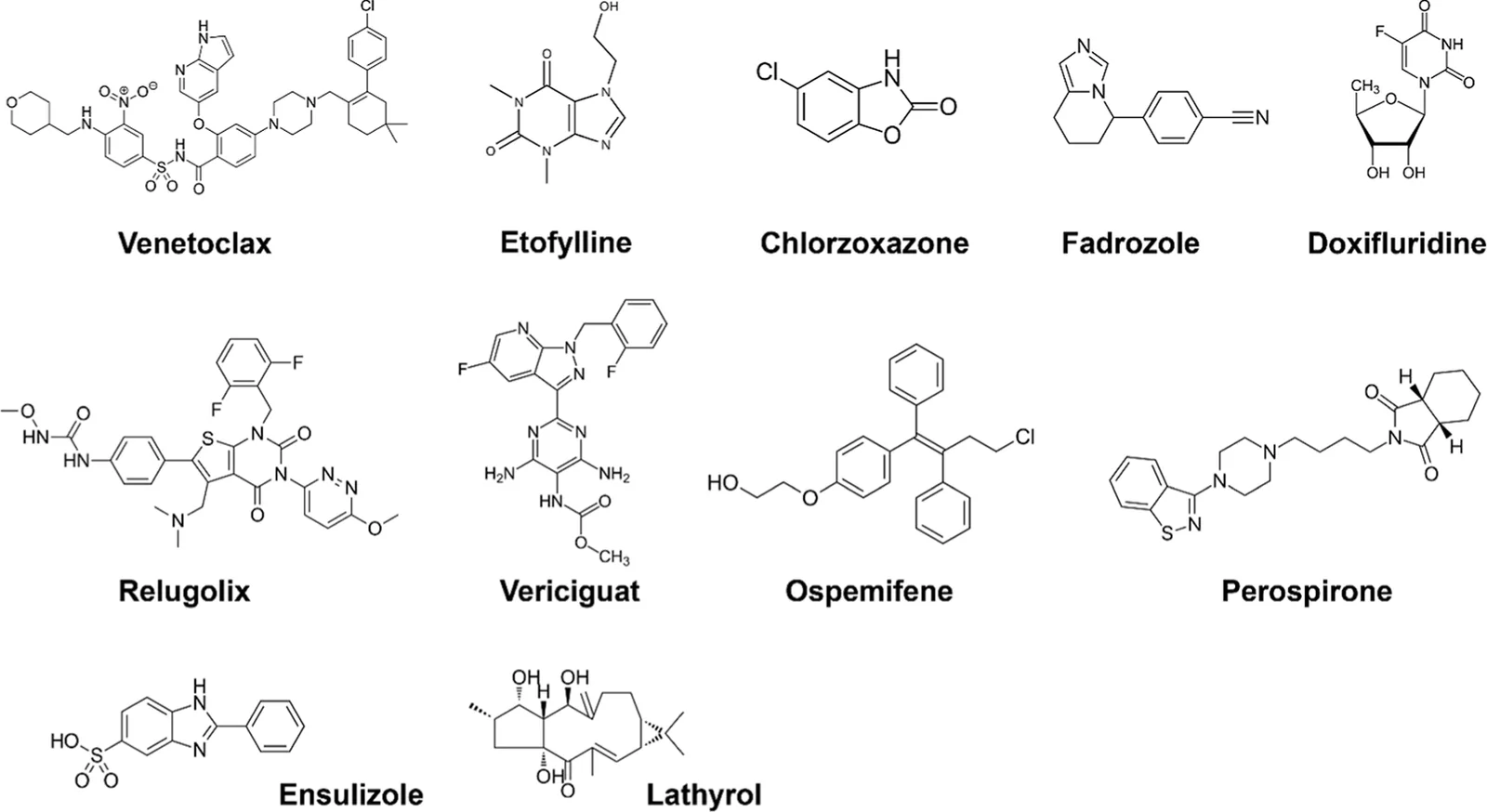
2D structures of selected FDA-approved compounds with Venetoclax as the reference molecule.

The NRI-based analysis was incorporated to provide a trajectory-level mechanistic interpretation that is not captured by static docking scores or end point binding free-energy estimates. While docking and MM/GBSA can prioritize compounds according to pose compatibility and relative energetic favorability, they do not directly describe how ligand binding reshapes residue-level communication within BCL-2 during the simulations. By extending NRI to include both protein residues and ligand heavy atoms, we were able to learn dynamic residue–residue and residue–ligand coupling patterns from the MD trajectories. This allowed candidate compounds to be evaluated not only by their predicted binding strength, but also by the extent to which they reproduced the dynamic interaction architecture observed for the reference inhibitor Venetoclax. Therefore, NRI served as an additional mechanistic filter to distinguish ligands that merely occupied the BCL-2 binding groove from those that induced a more reference-like dynamic interaction network. The NRI model utilizes a variational graph autoencoder architecture to recreate the trajectories of MD simulations by learning latent contact maps between protein residues and ligand heavy atoms. While previous NRI models focused on understanding protein dynamics in apo and holo forms by tracking the carbon alpha atoms of amino acids, we have extended the NRI model to track both carbon alpha atoms and ligand heavy atoms. This enhancement allows us to quantify ligand–protein interactions, protein stability, and interactions between ligand heavy atoms. By training the NRI model to mimic MD trajectories, we obtain a latent interaction matrix *z*_*ij*_ (0 ≤ *z*_*ij*_ ≤ 1) that quantifies the contribution of atom *j* to the dynamics of atom *i*. The protein–ligand interaction energy is evaluated using the matrix *z*_*ij*_ with details provided in the Methodology section. We trained 10 NRI models for each identified candidate compounds and Venetoclax, using the same hyperparameters for the variational graph autoencoder models. Figure 3A illustrates the interaction matrices *z*_*ij*_ for all ligands with a better efficiency score than Venetoclax, Relugolix as the lowest MM/GBSA score compound, and the reference compound. Etofylline’s heavy atoms significantly affect residue dynamics, according to the protein–ligand interaction maps, which results in a higher normalized protein–ligand interaction energy than any of the other compounds that were evaluated. Further demonstrating its robust binding potential, Relugolix, which was chosen as the option with the lowest MM/GBSA binding energy, also has a greater protein–ligand interaction energy than Venetoclax (except for residue 81). The contact maps of BCL-2 in complex with Etofylline, Relugolix, and Venetoclax are shown in Figure 3B–D, respectively. Etofylline forms a large number of interactions with the BCL-2 protein in spite of its comparatively small molecular size. However, the concentrated residue–residue interactions that are primarily seen between residues 70 and 100 suggest that it is unable to appreciably stabilize the overall BCL-2 structure. Relugolix, on the other hand, has a contact pattern that is quite similar to Venetoclax’s, indicating a comparable binding mechanism. However, in some areas, Relugolix creates fewer intraprotein interactions in BCL-2 than Venetoclax, which would indicate a marginally diminished stabilizing impact after binding.

**Figure 3:**
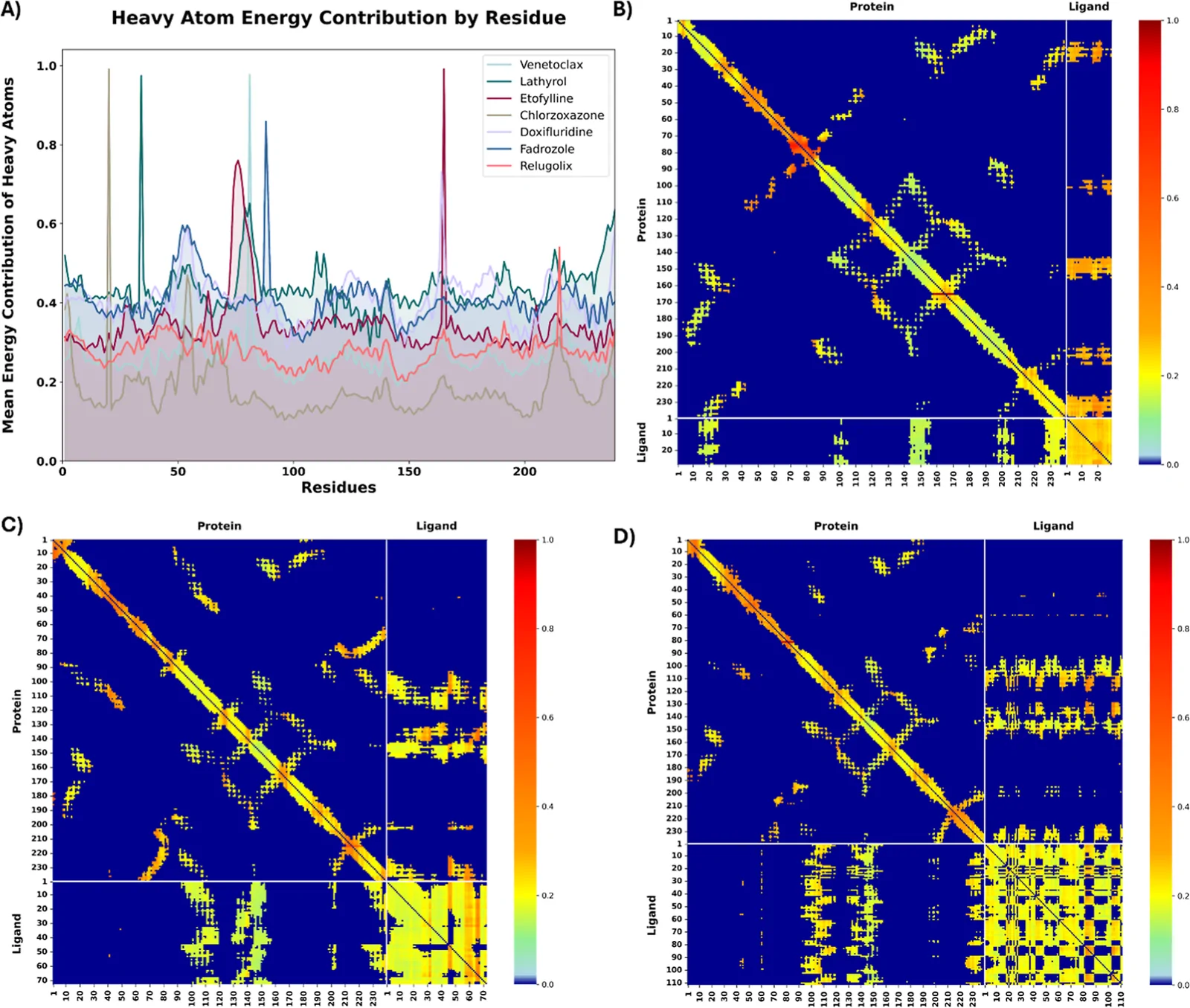
Results of the NRI model are shown as follows: (A) the mean energy contribution of the heavy atoms of all ligands with a better efficiency score than Venetoclax, Relugolix as the lowest MM/GBSA score compound, and Venetoclax as reference with BCL-2 protein residues; (B) a contact map of BCL-2 with Etofylline; (C) Relugolix, and (D) Venetoclax.

### 2.2 Machine Learning-Guided Prioritization of FDA-Approved BCL-2 Candidates

An ML-based prioritization branch was also developed alongside the NeuralPlexer-guided workflow to provide an orthogonal framework for candidate selection. NeuralPlexer offers a structure-aware route for modeling ligand-specific BCL-2 complexes, but its ranking of compounds is inherently linked to generated poses and subsequent physics-based evaluation. By contrast, Chemprop provides ligand-based activity prediction directly from molecular graphs and is therefore independent of explicit complex generation. The integration of these complementary approaches was intended to minimize reliance on a single computational paradigm, improve the robustness of hit prioritization, and reveal candidates supported by both structure-based and data-driven evidence. In this way, the ML-guided arm served not only as an additional screening route, but also as an internal cross-validation strategy prior to biochemical and cellular testing.

As described in the Methods section, we also trained a Chemprop^17^ model to predict pIC_50_ values using a data set of 1067 known BCL-2 inhibitors from ChEMBL (https://www.ebi.ac.uk/chembl/) with experimentally determined activities. Model training was performed using 5-fold cross-validation, with RMSE selected as the primary optimization metric. Over 50 training epochs, the model showed progressive improvement in both RMSE and *R* ^2^ across the validation sets. The best-performing fold achieved a validation RMSE of 0.636 and an *R* ^2^ of 0.889, indicating strong agreement between predicted and experimental pIC_50_ values. This optimal performance, reached at epoch 47, suggests that the model generalizes well across structurally diverse BCL-2 inhibitors. This validation performance was consistent with the results obtained on the independent test set, where the final model achieved an RMSE of 0.748 and an *R* ^2^ of 0.858, supporting its robustness and predictive reliability. The reproducibility of these results across cross-validation folds further indicates that the Chemprop architecture is stable for this regression task. Together, these metrics suggest that the model is sufficiently accurate for downstream applications such as virtual screening of FDA-approved compounds and falls within a reasonable range for ligand–target affinity prediction. Based on the predicted pIC_50_ values, compounds estimated to be more potent than Venetoclax were selected for further evaluation. Because pIC_50_ values can be influenced by molecular size, ligand efficiency was also calculated to enable size-normalized comparison of compound potency. Given the relatively high molecular weight of Venetoclax, all selected compounds showed better ligand efficiency values, thereby prioritizing smaller molecules with favorable predicted potency and allowing a more balanced comparison.

GNINA,^18^ which incorporates convolutional neural network (CNN)-based scoring, was then used to dock the selected compounds into the BCL-2 binding site. The resulting poses were filtered using CNN affinity scores, and only compounds with values greater than 6.0 were retained, yielding a total of 10 candidates. Notably, when normalized for molecular size, all 10 compounds exhibited CNN-based ligand efficiency values higher than that of Venetoclax, suggesting favorable binding potential relative to their size. These top-ranked compounds were subsequently evaluated by 3 independent replicates of 200 ns all-atom MD simulations, from which 2000 trajectory frames were collected for each simulation (i.e., 6000 frames per protein–ligand complex). Binding free energies were then estimated using the MM/GBSA method implemented in the Schrödinger Prime module. To further assess their potential anticancer relevance, the 10 candidates were analyzed using the MetaCore platform, which integrates cheminformatics and systems biology for functional prediction. Based on structural features and predicted interaction patterns, 6 of the 10 compounds showed anticancer activity scores above 0.5, the default MetaCore threshold for probable anticancer potential. Among these candidates, Baricitinib, Trazodone, and Buspirone displayed strong CNN affinity scores (≥6.5), high predicted pIC_50_ values (≥9.1), and anticancer activity scores of at least 0.8, consistent with favorable binding, predicted potency, and potential therapeutic relevance. In parallel, Lanraplenib, Trazodone, and Cobimetinib yielded the most favorable MM/GBSA binding free energies (−70.49, −62.29, and −58.34 kcal/mol, respectively), further supporting their potential as BCL-2 binders. Table 2 summarizes the predicted pIC_50_ values, CNN docking scores, MetaCore activity probabilities, and MM/GBSA free energies for all selected compounds, while their 2D structures are shown in Figure 4.

**Table 2:** Predicted pIC_50_ Values of FDA-Approved Compounds Obtained by ML Method with Venetoclax as the Reference Molecule, along with Their 3-Replicate Average MM/GBSA Scores, CNN Affinity Scores, Anticancer Activity Scores, and Ligand Efficiency Statistics

compound	predicted pIC_50_	average MM/GBSA (kcal/mol)	CNN affinity	anticancer activity score	ligand efficiency (kcal/mol)
Venetoclax	9.08	–132.14	9.33	0.72	–2.17
Lanraplenib	9.11	–70.49	6.82	0.66	–2.14
Trazodone	9.1	–62.29	6.72	0.82	–2.40
Cobimetinib	9.46	–58.34	7.59	0.63	–1.94
Clozapine *N*-oxide	9.09	–57.06	6.92	0.45	–2.38
Hexetidine	9.64	–54.96	6.67	0.27	–2.29
Buspirone	9.62	–54.39	7.19	0.82	–1.94
Tosufloxacin	9.16	–48	6.89	0.07	–1.66
Piperaquine	10.06	–41.91	7.96	0.68	–1.13
Baricitinib	9.33	–37.12	6.59	0.8	–1.43
Vildagliptin	9.09	–27.99	6.54	0.31	–1.27

**Figure 4:**
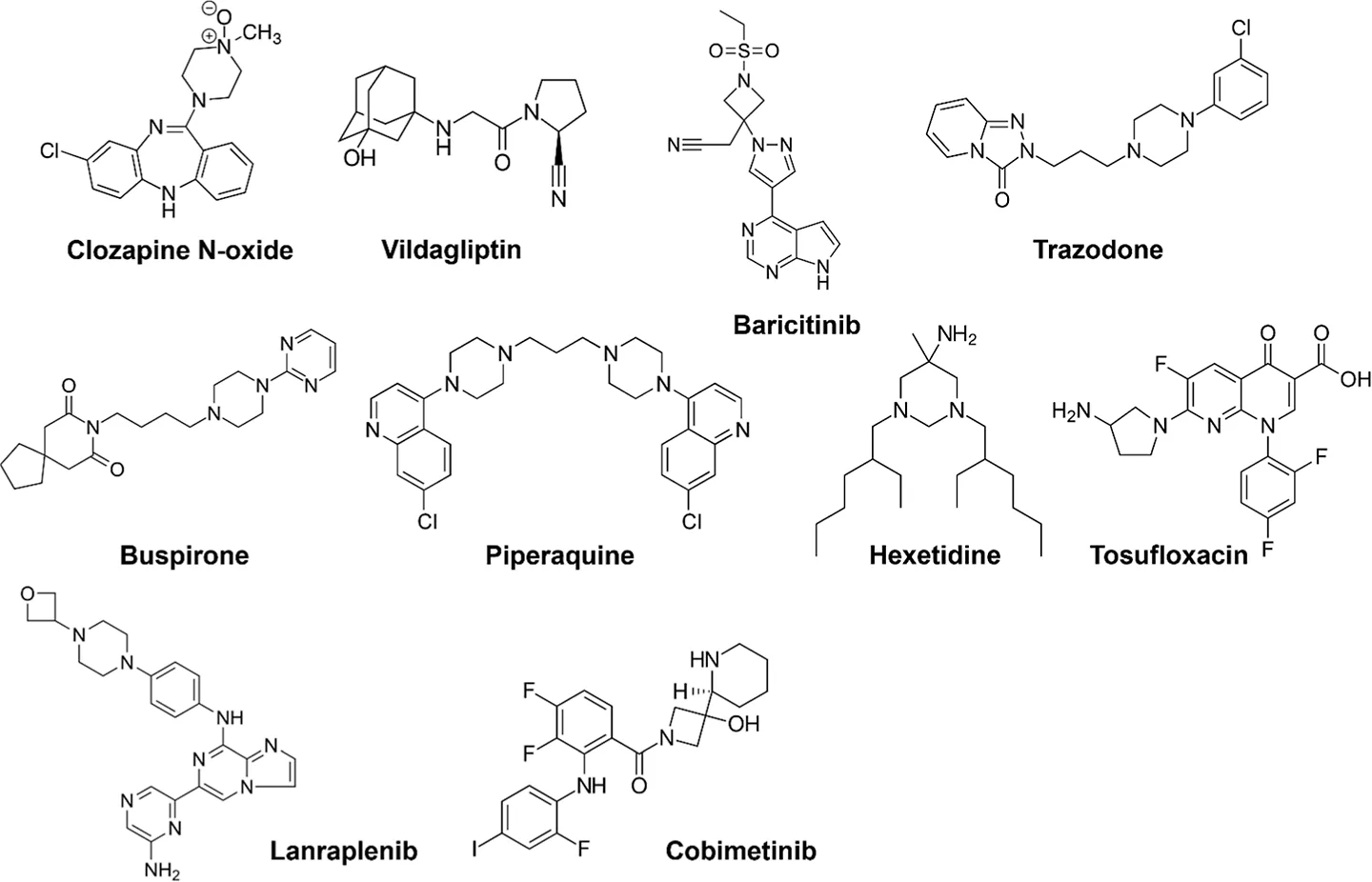
2D structures of FDA-approved compounds selected through the ML-based prioritization workflow.

To determine whether this ML-guided strategy could identify potent BCL-2 candidates complementary to those prioritized by the NeuralPlexer-based workflow, we compared the outputs of the two pipelines. Both the NeuralPlexer-based and Chemprop-guided approaches identified some of the compounds with potential BCL-2 inhibitory activities. These selected candidates were then prioritized for experimental follow-up and 12 of them subjected to in vitro enzyme inhibition and cell viability assays to assess their biological activity and therapeutic potential.

### 2.3 Experimental Validation of Prioritized BCL-2 Candidates

A time-resolved fluorescence resonance energy transfer (TR-FRET) assay was performed to assess whether the selected candidate compounds could disrupt the interaction between BCL-2 and its binding ligand. This assay provided a biochemical readout of inhibitory activity by measuring the extent to which each compound interfered with ligand engagement. Initial screening of 12 compounds showed that 4 molecules were able to compete with the known BCL-2 inhibitor, indicating measurable inhibitory activity. In most of the cases, inhibition of BCL-2–ligand binding increased with compound concentration, consistent with a concentration-dependent effect. Among the tested molecules, Vericiguat, Trazodone, Relugolix, and Chlorzoxazone produced the strongest inhibition at 100 μM (Figure 5). In contrast, the remaining 8 compounds showed small or no detectable inhibition and did not display a meaningful concentration-dependent response.

**Figure 5:**
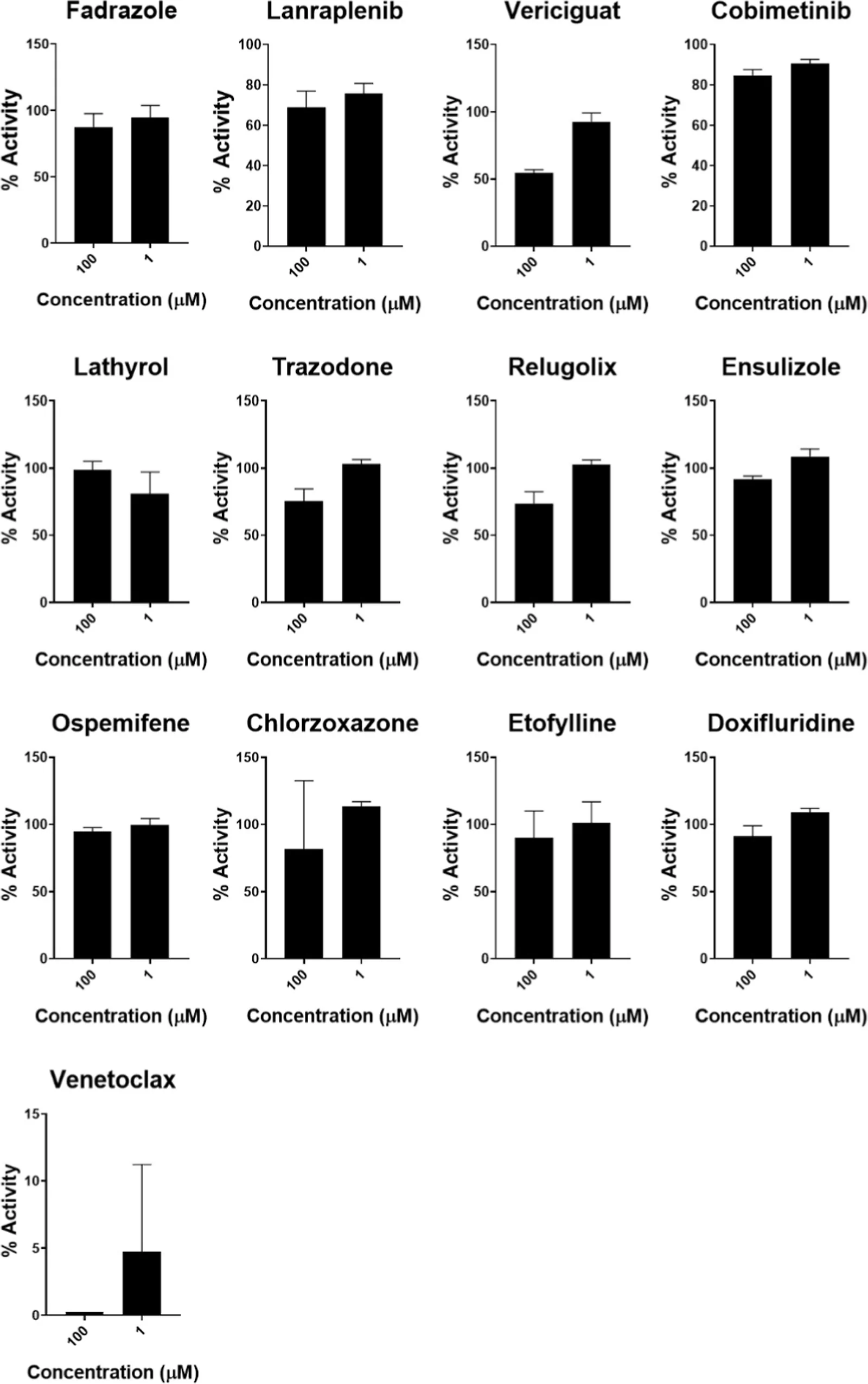
Inhibitory activity of the 12 selected hit compounds, with Venetoclax included as a positive control. Compounds were evaluated at 100 μM and 1 μM, and percentage inhibition was calculated using the equation described in the Materials and Methods section.

Based on the initial screening results, four compounds, Vericiguat, Lathyrol, Relugolix, and Chlorzoxazone, were selected for more detailed evaluation across five concentration levels (Figure 6). Although Trazodone showed notable activity in the first-pass assay, Lathyrol was prioritized for dose–response analysis because its initial profile suggested the possibility of inhibitory activity at lower concentrations. For this reason, Lathyrol was advanced to broader concentration-range testing in place of Trazodone. This second-stage analysis enabled a more refined comparison of inhibitory potency among the selected hits. To examine whether these biochemically active compounds also affected cancer cell growth, they were further tested in LN-18 glioma cells using a 24 h MTT cell viability assay. Treatment with the selected compounds led to reduced cell proliferation, providing preliminary cellular support for their predicted BCL-2 inhibitory activity (Figure 7). Taken together, the TR-FRET and cell viability results strengthened the prioritization of these compounds, particularly those that demonstrated both direct inhibition of BCL-2–ligand binding and measurable antiproliferative effects in cancer cells.

**Figure 6:**
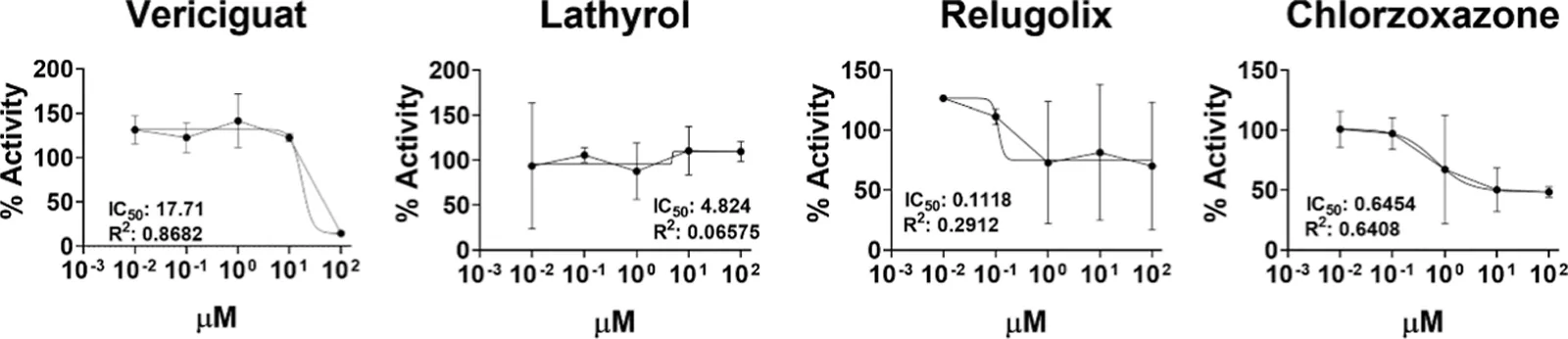
Maximum percentage inhibitory activity of four hit compounds across the tested concentration range (100 μM to 10 nM), together with the corresponding IC_50_ values determined after 3 h of incubation.

**Figure 7:**
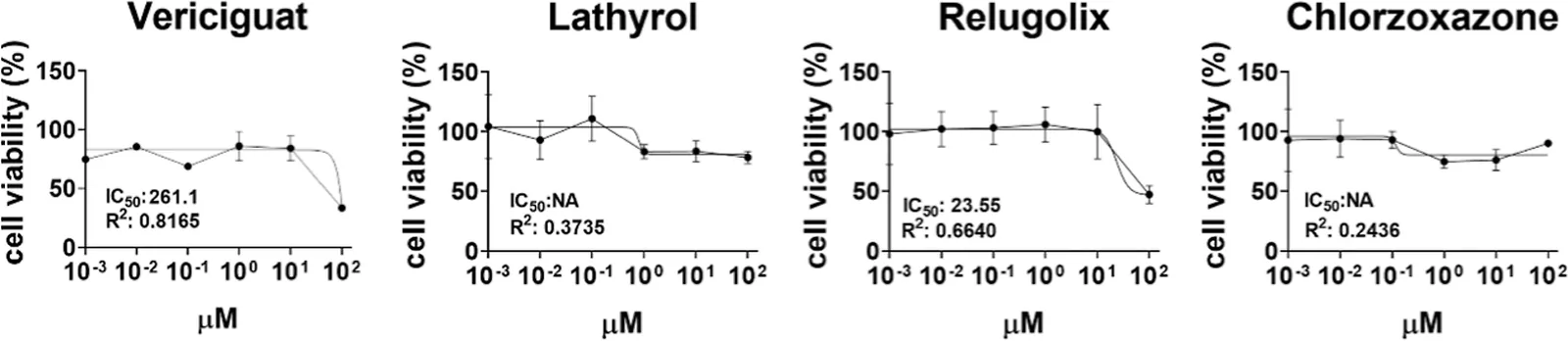
MTT-based cell proliferation assay of compounds that showed inhibitory activity. Each compound was tested over a concentration range from 100 μM to 1 nM. Cell viability measurements were performed after 24 h of treatment in LN-18 glioma cells.

Among the four compounds (Vericiguat, Lathyrol, Relugolix, and Chlorzoxazone) evaluated in the TR-FRET assay, Relugolix showed the strongest inhibitory activity, with an IC_50_ value of 0.11 μM, although the relatively low R^2^ value (0.29) suggests some uncertainty in the curve fit. Chlorzoxazone also demonstrated substantial BCL-2 inhibition, with an IC_50_ of 0.65 μM, whereas Vericiguat was less potent, with IC_50_ values of 17.71 μM. In terms of dose–response fitting, Vericiguat and Chlorzoxazone yielded the highest *R* ^2^ values (0.87 and 0.64, respectively), while the fits for Lathyrol and Relugolix showed greater variability (*R* ^2^ = 0.07 and 0.29, respectively). In the LN-18 glioma cell viability assay, Relugolix emerged as the most active compound, reducing cell viability with an IC_50_ of 23.55 μM and a reasonable curve fit (*R* ^2^ = 0.66), in agreement with its biochemical activity in the TR-FRET assay. By contrast, Vericiguat displayed a much weaker antiproliferative effect, with an IC_50_ of 261.1 μM, suggesting limited cytotoxic activity under the tested conditions. Chlorzoxazone and Lathyrol did not yield measurable IC_50_ values within the tested concentration range, indicating either weak or inconsistent effects on cell viability.

Taken together, these results suggest that Relugolix is a promising compound, showing BCL-2 inhibitory activity in the TR-FRET assay and a well-fitted antiproliferative response in cancer cells, although the TR-FRET IC_50_ estimate should be interpreted cautiously due to its relatively low R^2^ value.

## 3 Discussion

This study demonstrates the value of combining generative protein–ligand complex prediction, physics-based refinement, graph-based trajectory analysis, and experimental testing in a unified BCL-2 drug-repurposing workflow. Rather than relying on a single docking score or a purely ligand-based screening strategy, we designed a multimodal pipeline in which NeuralPlexer was first used to generate ligand-specific BCL-2 complex conformations, followed by docking refinement, MD simulations, MM/GBSA calculations, NRI, and *in vitro* validation. This integrated design enabled prioritization of compounds on the basis of structural plausibility, binding energetics, dynamic interaction patterns, and biological activity, thereby addressing several of the limitations that commonly affect conventional repurposing studies.

The computational components of this workflow should be interpreted within the known strengths and limitations of each method. Docking and grid-based refinement provided an efficient means to evaluate local compatibility of NeuralPlexer-generated poses, but their scoring functions remain approximate and may not fully capture receptor flexibility, long-time scale conformational adaptation, or solvent-mediated binding effects. MD simulations provided a dynamic description of ligand-bound BCL-2 complexes, but the 200 ns MD trajectories used here represent finite sampling windows rather than exhaustive exploration of the conformational landscape. Similarly, MM/GBSA calculations were used to support relative prioritization rather than to estimate exact thermodynamic binding affinities. For this reason, we did not base candidate selection on any single computational metric. Instead, computational confidence was assigned only when multiple orthogonal criteria converged, including structural plausibility, local pose stability, ligand efficiency, MD/MM-GBSA behavior, NRI-derived similarity to the Venetoclax interaction architecture, and subsequent biochemical and cellular validation.

A key contribution of the present work is the application of NeuralPlexer as the entry point for large-scale, structure-guided screening against BCL-2. In contrast to conventional docking workflows, which often assume a largely rigid receptor and rank compounds primarily through approximate scoring functions, NeuralPlexer generates ligand-specific complex conformations in a manner that is more compatible with protein–ligand structural cooperativity. In our study, this allowed the generation of bound-state models for a large FDA-approved drug set and yielded 1294 structurally acceptable complexes for downstream analysis. Importantly, the comparison with the co-crystallized Venetoclax–BCL-2 structure further supported the reliability of this approach, as NeuralPlexer reproduced the ligand pose with a binding-site RMSD of 0.35 Å while maintaining reasonable agreement at the protein level (Figure 8). Taken together, these findings support the use of NeuralPlexer-generated complexes as meaningful starting points for posthoc energetic and dynamical analysis in repurposing studies.

**Figure 8:**
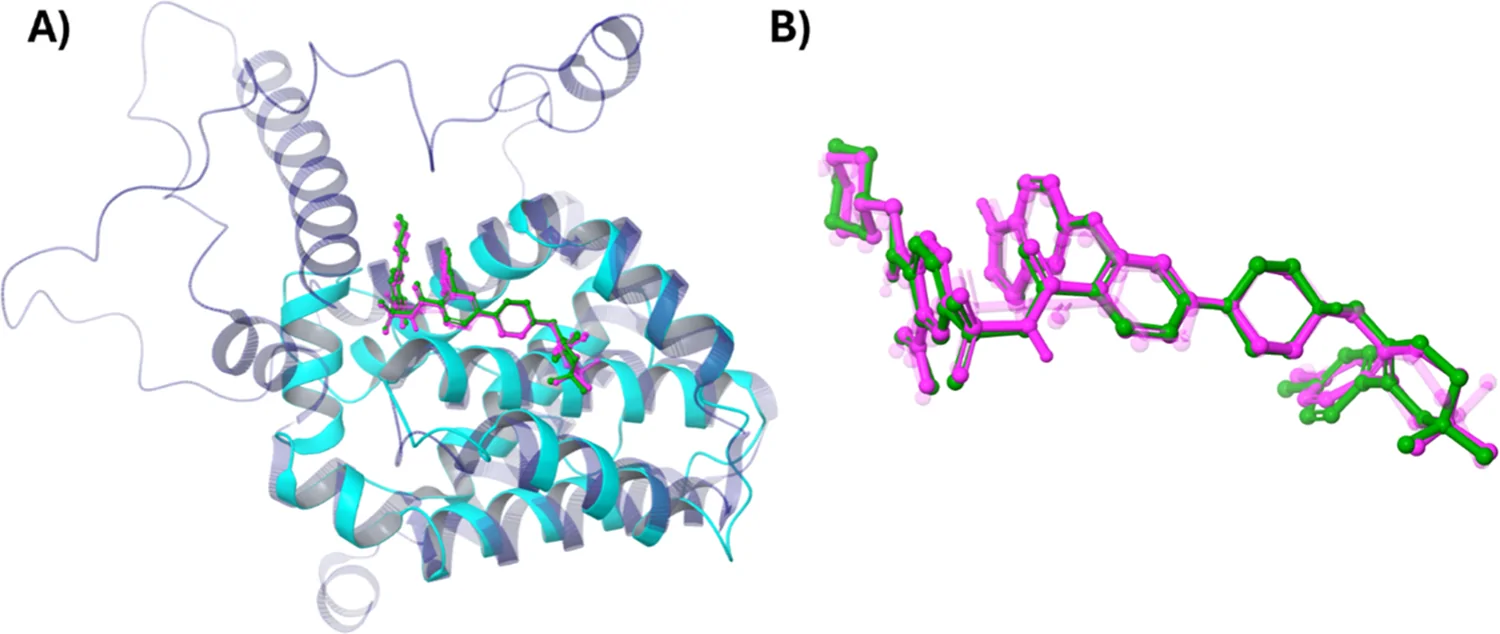
Comparison of the cocrystallized BCL-2 structure of Venetoclax (PDB: 6O0K) with NeuralPlexer predicted 3D structure. (A) The X-ray structure of BCL-2 is colored with cyan, while the NeuralPlexer predicted structure is represented with ice blue. (B) Venetoclax is colored as pink (X-ray conformer) and green (NeuralPlexer prediction).

The oral anticancer drug Doxifluridine had an average MM/GBSA score of −40.90 kcal/mol and a ligand efficiency score of −2.41 kcal/mol. It also has antiangiogenic properties. Even if it is not one of the best options, Doxifluridine is a worthwhile compound to look into further in the context of BCL-2-driven tumors due to its capacity to suppress angiogenesis and cause apoptosis in cancer cells. After Doxifluridine, the ligand efficiency of Fadrozole, an aromatase inhibitor, is −2.46 kcal/mol. It is approved to treat breast cancer and has an average MM/GBSA score of −41.88 kcal/mol. This implies that Fadrozole might be able to maintain a relatively modest molecular size while exhibiting a high affinity for binding to the BCL-2 protein binding site. Following Fadrozole, Chlorzoxazone has a slightly better ligand efficiency score of −3.17 kcal/mol. The possibility of the muscle relaxant chlorzoxazone as a BCL-2 inhibitor has not been previously explored. With a ligand efficiency score of −2.88 kcal/mol, etofylline, a phosphodiesterase inhibitor used to treat cerebrovascular diseases, shows promise as a strong and selective BCL-2 inhibitor.

The initial NeuralPlexer-guided prioritization identified several compounds with favorable ligand–efficiency profiles, including Etofylline, Chlorzoxazone, Fadrozole, and Doxifluridine. These molecules did not outperform Venetoclax in terms of average MM/GBSA binding free energy, which remained most favorable for the reference inhibitor, but they compared favorably after normalization for molecular size. This distinction is important. Average MM/GBSA values tend to favor larger ligands, whereas ligand efficiency can better reflect how effectively a compound engages the target relative to its size. From a repurposing and medicinal chemistry perspective, smaller compounds with favorable size-normalized binding characteristics may provide more tractable starting points for optimization, particularly when the reference ligand is relatively large, as in the case of Venetoclax. Accordingly, the NeuralPlexer branch did not simply identify compounds with strong nominal binding scores, but also highlighted chemically compact scaffolds with potentially useful binding efficiency. However, one of the most important findings of this study is that ligand efficiency alone was insufficient to define the most promising candidate. The NRI analysis added a mechanistic layer that changed how the top compounds were interpreted. By extending NRI to include both protein residues and ligand heavy atoms, we were able to move beyond static scoring and ask how each ligand altered the dynamic interaction network of BCL-2 during MD simulations. Etofylline, for example, produced strong residue-level influence in the learned interaction maps and displayed a high normalized protein–ligand interaction energy. Nevertheless, its contact pattern appeared more spatially concentrated, particularly in the region spanning residues 70–100, suggesting localized perturbation rather than broad stabilization of the bound protein state. Relugolix, in contrast, showed a contact pattern that more closely resembled that of Venetoclax, even though some intraprotein interactions remained weaker. This observation is important because it suggests that the most informative candidates are not necessarily those that maximize one energy-derived metric, but those that best reproduce the interaction architecture of a productive reference binder.

This mechanistic distinction helps explain why Relugolix emerged as the most compelling hit from the NeuralPlexer/NRI arm of the workflow. Relugolix was not the strongest compound by ligand efficiency, yet it combined several favorable properties. A comparatively strong MM/GBSA profile among the repurposed candidates, an NRI-derived interaction pattern that closely paralleled Venetoclax, and consistent performance in subsequent biochemical and cellular assays. In other words, its prioritization was driven by multiparameter convergence rather than by any single ranking criterion. This is an important aspect of the workflow, because one of the persistent problems in repurposing is overinterpretation of compounds selected only on the basis of docking, free-energy estimates, or ligand-based prediction. Here, Relugolix advanced because it was supported simultaneously by structure generation, energetic refinement, dynamic network analysis, and experimental follow-up.

Clinically, relugolix is an orally active gonadotropin-releasing hormone (GnRH) receptor antagonist currently approved for the treatment of advanced prostate cancer and, in combination with estradiol and norethindrone acetate, for the management of heavy menstrual bleeding associated with uterine fibroids and moderate to severe pain associated with endometriosis in premenopausal women.^20^ As such, its established clinical utility is rooted in endocrine modulation rather than in direct targeting of apoptotic machinery. This is an important distinction, because the canonical clinical example of BCL-2 inhibition remains venetoclax, which is specifically approved as a BCL-2 inhibitor in hematologic malignancies. In this context, the relevance of relugolix lies in the fact that, despite belonging to a therapeutically distinct class, it reproduced key Venetoclax-like interaction features within the BCL-2 binding environment and demonstrated convergent support across structure generation, energetic refinement, network-level analysis, and downstream experimental evaluation. Taken together, these observations raise the possibility that relugolix may possess off-target or secondary BCL-2-modulating potential, a hypothesis that warrants direct mechanistic validation through dedicated target-engagement and further apoptosis-focused studies.

The second major component of the study was the ML-guided prioritization branch based on Chemprop and GNINA. This arm served an important role because it provided an orthogonal, ligand-centric perspective that was independent of explicit NeuralPlexer complex generation. The Chemprop model achieved good predictive performance on known BCL-2 inhibitors, and the downstream GNINA/MM-GBSA/MetaCore pipeline identified several additional compounds with plausible BCL-2 activity, including Lanraplenib, Cobimetinib, Trazodone, Baricitinib, and Buspirone. While this branch did not ultimately yield the experimentally strongest candidate, it strengthened the overall study by reducing dependence on a single computational paradigm. In practical terms, the Chemprop-guided workflow functioned as an internal cross-check on the structure-based prioritization strategy and demonstrated that the proposed screening framework is not limited to one class of computational evidence. Notably, retrospective application of the Chemprop model to the four experimentally validated NeuralPlexer-derived hits (Vericiguat, Lathyrol, Relugolix, and Chlorzoxazone) yielded predicted pIC_50_ values of 6.69, 4.56, 6.50, and 7.84, respectively. This discrepancy suggests that a purely ligand-based screening strategy would likely have deprioritized these compounds, reinforcing the added value of the structure-based NeuralPlexer/NRI arm in surfacing candidates that a conventional QSAR-type model might overlook.

The experimental results suggest that the NeuralPlexer-guided workflow provided the more effective route for identifying compounds that translated into measurable biological activity. Among the compounds that showed the strongest initial TR-FRET inhibition, most were selected from the NeuralPlexer-based pipeline, and the final dose–response and cell-viability experiments further strengthened this trend, with Relugolix emerging as the potentially most convincing experimentally promising candidate and Chlorzoxazone and Vericiguat also showing biochemical activity. By comparison, the ML-guided branch contributed at least one promising candidate, Trazodone, in the first-pass assay, indicating that ligand-based prediction can still enrich the screening set with biologically relevant compounds. However, the fact that the most robustly supported compounds ultimately originated from the NeuralPlexer-guided arm suggests that explicit modeling of ligand-specific BCL-2 complexes, followed by MD/MM-GBSA and NRI-based dynamic analysis, may provide higher practical precision for selecting experimentally successful candidates in this target system.

At the same time, the current results do not suggest that Relugolix is superior to Venetoclax. Venetoclax remained stronger in average MM/GBSA binding energetics and continues to outperform the repurposed candidates under the tested conditions. The significance of Relugolix lies elsewhere: It represents an experimentally supported noncanonical scaffold that may engages BCL-2 and displays measurable cellular activity, despite not having been developed as a BCL-2 inhibitor. For this reason, Relugolix is best viewed not as a replacement for Venetoclax, but as a promising starting point for medicinal chemistry optimization. Its experimentally supported partial activity, combined with a dynamic interaction profile resembling that of Venetoclax, makes it a suitable scaffold for analog design aimed at strengthening target-specific contacts, improving potency, and refining selectivity.

The overall findings establish a useful conceptual framework for BCL-2-focused repurposing. The study shows that generative complex prediction can be combined with NRI-based dynamic analysis to prioritize compounds not only by static fit or predicted affinity, but also by their ability to reproduce interaction patterns associated with a productive reference inhibitor. The inclusion of an orthogonal Chemprop/GNINA branch further improves the robustness of candidate selection, while experimental follow-up provides an essential filter against computational false positives. In this context, the main advance of the work is not simply the identification of Relugolix, but the demonstration that structure-aware generation, dynamic graph inference, and ligand-based learning can be integrated into a practical workflow for repurposing against BCL-2. This strategy should be transferable to other targets for which ligand-induced conformational adaptation and residue-level communication are important determinants of productive binding.

From a translational perspective, the most immediate next step is the optimization of Relugolix-derived analogs guided by the structural and dynamical information generated here. Compounds derived from the Relugolix scaffold could be designed to strengthen the interactions that overlap with Venetoclax-like binding while compensating for the weaker intraprotein stabilization observed in the NRI and contact-map analyses. More broadly, the present results support the idea that repurposing campaigns should move beyond single-metric prioritization and toward integrated decision-making frameworks in which structure generation, dynamic interpretation, and experiment are explicitly linked. Within that broader framework, Relugolix emerges as the most convincing lead outcome of the current study and a reasonable starting point for future BCL-2 inhibitor development.

## 4 Conclusions

In this study, we established a multimodal computational-experimental workflow for BCL-2-focused drug repurposing that integrates NeuralPlexer-based protein–ligand complex generation, physics-based postprocessing, extended NRI, and an orthogonal ML-guided prioritization strategy. Applied to a library of 3094 FDA-approved compounds, this framework enabled large-scale generation of ligand-specific BCL-2 complex conformations, systematic filtering of candidate binders, and dynamic comparison of prioritized compounds against the reference inhibitor Venetoclax. By extending NRI to protein–ligand trajectories, we further moved beyond static scoring and incorporated residue-level dynamic interaction patterns into candidate selection, thereby providing an additional mechanistic layer for prioritization.

The results highlight two principal advances. First, the study demonstrates that NeuralPlexer can serve as a practical entry point for large-scale, structure-guided repurposing against BCL-2, generating chemically meaningful bound complexes suitable for downstream refinement and trajectory analysis. Second, the combination of NRI-based dynamic inference with conventional scoring metrics improved interpretation of the prioritized compounds by distinguishing candidates that merely scored favorably from those that more closely reproduced the interaction architecture of a productive BCL-2 binder. In parallel, the Chemprop/GNINA branch provided an orthogonal ligand-centric route for candidate prioritization, thereby increasing the robustness of the overall screening strategy and reducing dependence on a single computational paradigm. Among the evaluated compounds, Relugolix emerged as the most compelling experimentally supported hit. Its prioritization was not driven by a single metric, but by convergence across multiple layers of evidence, including favorable binding energetics, a Venetoclax-like NRI interaction signature, measurable BCL-2 inhibition in the TR-FRET assay, and antiproliferative activity in LN-18 glioma cells. These findings support Relugolix as a validated noncanonical BCL-2-interacting scaffold and a promising starting point for future optimization.

Taken together, this work shows that generative complex prediction, graph-based dynamic analysis, ligand-based ML, and experimental validation can be combined into a coherent and effective framework for structure-guided drug repurposing. Beyond the identification of Relugolix, the broader significance of the study lies in demonstrating a transferable strategy for targets in which ligand-induced conformational adaptation and residue-level communication are important determinants of productive binding. We anticipate that this integrated workflow will be useful not only for BCL-2-driven malignancies, but also for other therapeutic targets where static scoring alone is insufficient for reliable candidate prioritization.

## 5 Methods

### 5.1 Generation of BCL-2–Ligand Complex Conformations with Diffusion-Based Generative Model

In this study, we generated high-quality conformations of the BCL-2 protein and the 3094 FDA-approved drugs from the DrugBank database using the protein-ligand structure prediction tool, NeuralPlexer.^12^ Using protein sequences and ligand molecules as input, the deep generative model NeuralPlexer can accurately predict the 3D structures of protein–ligand complexes. The following are the main ways that NeuralPlexer^12^ creates the conformations: NeuralPlexer samples the 3D coordinates of the ligand atoms iteratively by means of a diffusion process. This enables the model to provide realistic and varied ligand conformations that meet important biophysical requirements. Also, the model uses a hierarchical multiscale architecture to improve the all-atom 3D coordinates step-by-step after predicting residue-level contact maps. NeuralPlexer is able to capture the intricate structural cooperativity between the ligand and protein as a result. NeuralPlexer’s diffusion mechanism is engineered to take into account crucial biophysical limitations such as lengths, angles, and steric conflicts. This aids in the generation of physiologically plausible ligand conformations by the model. NeuralPlexer can capture the conformational changes brought about by ligand binding since it is trained to sample the protein in both its ligand-bound and ligand-free states. An ensemble of predicted ligand conformations is produced by NeuralPlexer, so they can be sorted and chosen according to confidence scores or other standards to determine the most promising binding poses. The following particular settings that are applied when using NeuralPlexer: Produce 8 distinct forms for every protein–ligand complexes, divide the input into 4 segments to maximize memory utilization, 40 diffusion stages are needed to produce the final conformations, apply the sampling strategy of Langevin Simulated Annealing, find and manage connections between the ligand and the protein, then sort the produced conformations according to the scores for confidence. NeuralPlexer produced high-quality conformations, which served as the foundation for further computational investigation. This included calculations of binding free energy, molecular docking, and MD simulations. The success of this investigation was largely dependent on NeuralPlexer’s capacity to precisely capture the intricate structural cooperativity between the BCL-2 protein and small molecule ligands.

### 5.2 Glide-Based Pose Refinement and Binding Score Estimation

The protein–ligand complex conformations generated by NeuralPlexer provide valuable structural insight. However, estimation of binding affinity is required to assess their therapeutic potential more rigorously. To enable this step, we developed a Python script, which is available through our GitHub repository (https://github.com/DurdagiLab/Neuralplexer_Ligand_Scoring). The script first uses Maestro’s built-in tools to merge and prepare the protein–ligand complex structures generated by NeuralPlexer. A molecular docking grid is then defined by centering the grid box on the ligand centroid, thereby specifying the binding site for subsequent calculations. Glide is subsequently applied to estimate the Standard Precision (SP) docking score of each complex.^21^ In addition, Glide’s “refine only” mode is used to locally optimize the ligand conformation starting from the NeuralPlexer-generated pose, without performing extensive resampling, and to report the corresponding optimized SP docking score for each protein–ligand complex. By combining the robust scoring framework of Glide with the ligand-specific complex conformations generated by NeuralPlexer, this workflow enables efficient evaluation of the predicted binding affinities of candidate compounds. In this context, the “refine only” option provides a practical balance between computational cost and scoring reliability by allowing limited conformational adjustment while preserving the overall binding geometry predicted by NeuralPlexer. Overall, the Python-based workflow streamlines the screening process and supports high-throughput prioritization of large chemical libraries based on predicted binding affinity.

### 5.3 MetaCore Binary QSAR-Based Anticancer Activity Filtering

After SP docking scores had been obtained, compounds were grouped into individual *sdf* files according to their docking results, provided that the ligand conformation generated in Glide “refine only” mode did not deviate by more than 2 Å from the original NeuralPlexer pose. These files were then submitted to the MetaCore platform for screening with the “cancer therapeutic activity prediction QSAR” module, which estimates the likelihood that a compound possesses anticancer properties. In this framework, therapeutic activity values (TAVs) are assigned by comparing the query compounds with compounds known to have strong anticancer activity.^22,23^ TAV scores are normalized between 0 and 1, and values above 0.5 are considered indicative of potential anticancer activity. The MetaCore cancer-QSAR model was developed using a training set of 886 compounds and a defined descriptor set, achieving a sensitivity of 0.95, specificity of 0.92, accuracy of 0.93, and a Matthews correlation coefficient (MCC) of 0.87. Validation on an external test set of 167 compounds yielded a sensitivity of 0.89, specificity of 0.83, accuracy of 0.86, and an MCC of 0.72. Based on these criteria, a TAV threshold of 0.5 was applied for candidate selection, and among the compounds that passed this cutoff, the 10 molecules with the most favorable docking scores were retained for further analysis.

### 5.4 Atomic-Level Refinement of Prioritized Protein Structures

Protein structures associated with compounds that satisfied the binary QSAR cutoff of 0.5 were further refined using ModRefiner,^24^ a high-resolution protein structure refinement method. ModRefiner can improve structural models at atomic resolution starting from Cα traces, backbone-only models, or full-atom structures. During refinement, both backbone and side-chain atoms remain flexible, and the conformational search is guided by a combination of physics-based and knowledge-based force fields. The method can also incorporate a reference structure to improve backbone topology, side-chain orientation, and hydrogen-bonding geometry, thereby bringing the model closer to a physically realistic native-like state. In addition, the stand-alone implementation supports *ab initio* full-atom relaxation without constraining the refined model to the starting or reference structure. In the present study, this refinement step was included to correct potential local structural inaccuracies, particularly proline-related geometric errors, in the NeuralPlexer-generated protein conformations.

### 5.5 Protein Preparation for Molecular Simulations

To prepare the protein, the Protein Preparation Tool^25^ was used. This included reassembling disulfide bonds, adding hydrogen atoms, forming zero-order bonds with metals, and allocating bond orders. With the help of PROPKA, the target protein’s residues’ protonation states at physiological pH were assigned, and the OPLS3e force field^26^ was used to reduce the side chain atoms.

### 5.6 All-Atom MD Simulations of BCL-2-Ligand Complexes

All-atom MD simulations were subsequently performed, following the preparation of the selected compounds and Venetoclax as the reference molecule. The protein–ligand complex was positioned in a solvation box with the TIP3P^27^ water model in the simulations, which were run using the Desmond.^28^ A buffer zone measuring 10 Å in an orthorhombic box encircled the compound. Protonation states were assigned at pH 7.4 using Epik based on predicted p*K*_a_ values. The system was neutralized with Na^+^ counterions and brought to physiological ionic strength (0.15 M NaCl). Ligand force-field parameters were assigned using the OPLS3e^26^ force field, which provides atom-type coverage for a broad range of drug-like heterocycles without requiring custom parametrization. Each system was subjected to the Desmond default relaxation protocol prior to production simulation. This protocol consists of five sequential stages: (i) energy minimization of solvent and ions with solute restrained (2000 steps); (ii) 12 ps NVT MD at 10 K with solute restrained; (iii) 12 ps NPT MD at 10 K with solute restrained; (iv) 12 ps NPT MD at 310 K with solute restrained; and (v) 24 ps NPT MD at 310 K with all restraints released, allowing the full system to equilibrate prior to production. Production MD was run for 200 ns per compound under the *NPT* ensemble at 310 K and 1.01325 bar, controlled by the Nosé-Hoover thermostat^29^ and Martyna–Tobias–Klein barostat,^30^ respectively. Equations of motion were integrated using the RESPA integrator with 2 fs (bonded), 2 fs (short-range nonbonded), and 6 fs (long-range nonbonded) time steps. Short-range electrostatic and van der Waals interactions were evaluated with a 9 Å cutoff; long-range electrostatics were treated by the particle mesh Ewald (PME) method^31^ under periodic boundary conditions. Each compound was simulated as 3 independent replicate simulations. A total of 2000 frames were saved at equal intervals (one frame per 100 ps) from each 200 ns production trajectory for subsequent analysis.

### 5.7 MM/GBSA Binding Free-Energy Calculations

Binding free energies were estimated using the MM/GBSA approach implemented in the Prime^32^ module of Maestro (Schrödinger). For each compound, 2000 evenly spaced snapshots were extracted from the full 200 ns production trajectory (one frame per 100 ps), covering the entire simulation to ensure representative conformational sampling. The stability of binding-energy profiles across the trajectory was used as an indicator of convergence. Three independent MD replicates were performed. The VSGB 2.0 ^33^ implicit solvation model was applied, with an external dielectric constant fixed at 80 and an internal dielectric constant that varies between 1.0 and 4.0 under the OPLS3e force field,^26^ consistent with standard practice for protein–ligand MM/GBSA calculations. Mean binding free energies and standard deviations are reported for each compound.

The MM/GBSA values were interpreted as comparative binding-energy estimates for candidate prioritization rather than as rigorous absolute binding free energies. Because MM/GBSA relies on an implicit solvent approximation and does not fully account for exhaustive conformational sampling, entropic contributions, or all solvent-mediated effects, the calculated values were used primarily to rank compounds within the same computational protocol and force-field environment. To reduce size-dependent bias, particularly in comparisons with the large reference inhibitor Venetoclax, binding-energy values were additionally evaluated in terms of ligand efficiency normalized by the number of non-hydrogen atoms. Accordingly, MM/GBSA was not used as an isolated determinant of activity, but as one component of a broader decision framework that also incorporated docking-pose stability, MD-derived interaction behavior, NRI-based dynamic coupling patterns, ML-guided prioritization, and experimental assay outcomes.

### 5.8 NRI-Based Analysis of Protein–Ligand Dynamic Interaction Networks

It is crucial to comprehend the intricate relationships found in nature, particularly as they relate to physical dynamical systems. Complex interacting atoms are produced by MD simulations, and conventional examination of pairwise relationships between residues requires the assumption of linear correlation. The NRI model has an unsupervised variational graph-based autoencoder architecture and have been proposed for the investigation of complex interactions in dynamical systems, particularly in protein dynamics.^15,16^ By comprehending the nonlinear linkages as an interaction graph or latent representation *z*_*ij*_, which represents the strength of the interaction between residues *i* and *j*, the NRI model seeks to recover dynamic trajectories. To evaluate protein–ligand energy scores, we use the formula

$$E_{z}=\frac{1}{n_{ligand}}\sum_{i=1}^{n_{residue}}\sum_{j=n_{residue}+1}^{n_{residue}+n_{ligand}}z_{ij}\delta_{ij}$$

where *n*_residue_ and *n*_ligand_ represent protein residue number and ligand heavy atom number, *z*_*ij*_ is the learned interaction matrix and δ_*ij*_ = 1 for ∥ ***x***_*i*_^0^ – ***x***_*j*_^0^∥ ≤ 12 Å and δ_*ij*_ = 0 for ∥ ***x***_*i*_^0^ – ***x***_*j*_^0^∥ > 12 Å. We followed the literature to select 12 Å as the threshold distance while evaluating the energy score *E*_z_.^15,16^ Higher energy scores mean that the ligand stabilizes the protein more strongly. In other words, *E*_z_ measures how much ligand’s heavy atoms affect the residue dynamics on average.

In this study, we extended the original NRI framework to reconstruct not only residue trajectories but also ligand trajectories, thereby enabling simultaneous learning of residue–residue, residue-ligand, and ligand–ligand interactions within protein–ligand complexes. Using this approach, we analyzed 2000-frame MD trajectories for a total of 11 BCL-2-ligand systems. The mathematical formulation and original implementation of the NRI model have been described previously.^15,16^ Here, we adapted the published source code to support protein–ligand trajectory analysis. To facilitate automation and reproducibility, the revised NRI workflow for protein–ligand complexes has been made available through our GitHub repository (https://github.com/DurdagiLab/Automate-Neural-relational-inference-NRI-).

This implementation automatically organizes the trajectory files, initializes model training, analyzes the resulting interaction maps, and exports the outputs as plots and CSV files.

### 5.9 Chemprop Model Development for pIC_50_ Prediction

The parallel ligand-based arm, combining a Chemprop message-passing neural network for pIC_50_ prediction with GNINA docking, was included as an orthogonal validation strategy. Chemprop operates directly on molecular graphs without relying on predefined fingerprints, allowing it to capture structure–activity relationships from training data in a manner that is complementary to the physics-based scoring used in the primary structure-based pipeline. Convergence between the two independent arms, structure-based and ligand-based, provides higher confidence that identified hits are not artifacts of any single scoring scheme.

A data set of 1067 compounds with experimentally determined pIC_50_ values was obtained from the ChEMBL database. (https://www.ebi.ac.uk/chembl/) Each molecule was represented by its canonical SMILES string, and activity data reported as IC_50_ values in molar units were converted to pIC_50_ using the relation pIC_50_ = −log_10_(IC_50_ [*M*]). To ensure reproducibility, the data set was split into training (80%), validation (10%), and test (10%) sets using a fixed random seed (4523). Model development was carried out with Chemprop, a graph-based deep learning framework that employs message-passing neural networks (MPNNs) to learn directly from molecular graph representations.^17^ The model was configured for regression to predict pIC_50_ values, with key hyperparameters including a message-passing depth of 3, two feed-forward hidden layers of size 300, and a batch size of 50. Training was performed for 50 epochs using 5-fold cross-validation, with mean squared error (MSE) as the optimization loss function and a learning-rate schedule ranging from 1 × 10^–4^ to 1 × 10^–3^. Model performance was monitored primarily using root-mean-square error (RMSE), while *R* ^2^ was also reported as an additional measure of predictive accuracy. The final trained model was then applied to a data set of FDA-approved drugs provided in CSV format with canonical SMILES strings, and Chemprop’s command–line interface was used to generate predicted pIC_50_ values for downstream analysis. To support reproducibility, the SMILES-based data set splits and model checkpoints were retained.

### 5.10 GNINA Docking and CNN-Based Prioritization of ML-Selected Compounds

Molecular docking was performed using GNINA^18^ an AutoDock Vina–based method that incorporates convolutional neural network (CNN) scoring to improve binding affinity prediction. The prepared crystal structure of human BCL-2 (PDB ID: 6O0K) was used as the receptor, and ligand structures were generated from SMILES strings using LigPrep to obtain 3D conformers and protonation states appropriate for physiological pH. The binding pocket was defined by a grid box centered on the known BCL-2 binding site and sized to encompass the key interacting residues. Molecular docking simulations were carried out with GNINA v1.0 using the default CNN scoring model and an exhaustiveness value of 8, generating multiple poses for each ligand. For each compound, the pose with the highest CNN affinity score was selected for further analysis, while both CNN-based affinity scores and conventional Vina scores were recorded. Compounds with CNN affinity scores greater than 6.0 were retained as potential high-affinity binders. In addition, CNN-based ligand efficiency values were calculated by normalizing the predicted binding score by the number of nonhydrogen atoms in each molecule. The resulting top-ranked candidates were then prioritized for MM/GBSA binding free-energy calculations and MD simulations.

### 5.11 TR-FRET Assay for Evaluation of BCL-2 Inhibition

The BCL-2 TR-FRET Assay Kit (50222-1, BPS, USA) was used to measure the inhibition of BCL-2 (B-cell lymphoma 2) binding to its ligand in the presence of BCL-2 inhibitory molecules in a homogeneous 96-well format. The assay protocol for TR-FRET analysis was performed based on the suggestions of the manufacturer. Briefly, a sample containing antihis terbium-labeled donor, dye-labeled streptavidin acceptor, BCL-2 protein, peptide ligand, and each inhibitor were incubated at room temperature for 3 h. All samples and controls were examined in duplicate. Venetoclax was used as a positive control drug. After 3 h of incubation at room temperature, the plates were read on a microplate reader (Varioskan Lux, Thermo Fisher, USA) with 340 ± 20 nm laser excitation, a first emission filter at 620 ± 10 nm, and a second emission filter at 665 ± 10 nm. The percentage inhibitory activity of tested molecules was calculated by

$$\%\;activity=\frac{FRETs-FRETneg}{FRETp-FRETneg}\times 100\%$$

where FRETs, FRETneg, and FRETp are sample FRET, negative control FRET, and positive control FRET, respectively.

### 5.12 Cell Viability Assay in LN-18 Glioma Cells

LN-18 (#CRL-2610, ATCC, USA) glioma cell line was used for cell culture experiments. Cells were seeded with high glucose Dulbecco’s Modified Eagle Medium (DMEM) medium (Capricorne) supplemented with 10% fetal bovine serum (FBS) (Gibco) and 1× penicillin/streptomycin (Multicell). 24 h prior to molecule treatment, 10,000 cells were seeded into each well of 96-well cell culture plates. Values of half-maximal inhibitory concentration (IC_50_) were determined by 3-(4,5-dimethylthiazol-2-yl)-2,5-diphenyltetrazolium bromide (MTT) cell proliferation assays. Different concentrations of molecules ranging between 100 μM and 1 nM were tested. Absorbance was measured at 570 nm with microplate reader (Varioskan Lux, Thermo Fisher, USA), and IC_50_ values were calculated by dose–response inhibition curves and nonlinear regression analysis on GraphPad Prism 8 software. For cell proliferation assays, we performed 24 h experiments performed in triplicate.

## Data Availability

The simulations were conducted with Desmond program and the MD data collected as trajectory files, and NeuralPlexer structures of top candidates were made available via the following repository: https://zenodo.org/records/19727467.
